# Aluminium is essential for root growth and development of tea plants (*Camellia sinensis*)

**DOI:** 10.1111/jipb.12942

**Published:** 2020-05-15

**Authors:** Lili Sun, Mengshi Zhang, Xiaomei Liu, Qianzhuo Mao, Chen Shi, Leon V. Kochian, Hong Liao

**Affiliations:** ^1^ Root Biology Center, College of Resources and Environment Fujian Agriculture and Forestry University Fuzhou 350002 China; ^2^ Vector‐Borne Virus Research Center Fujian Agriculture and Forestry University Fuzhou 350002 China; ^3^ Global Institute for Food Security University of Saskatchewan Saskatoon S7N 4J8 Canada

**Keywords:** aluminium, essential, meristem, root, tea

## Abstract

On acid soils, the trivalent aluminium ion (Al^3+^) predominates and is very rhizotoxic to most plant species. For some native plant species adapted to acid soils including tea (*Camellia sinensis*), Al^3+^ has been regarded as a beneficial mineral element. In this study, we discovered that Al^3+^ is actually essential for tea root growth and development in all the tested varieties. Aluminum ion promoted new root growth in five representative tea varieties with dose‐dependent responses to Al^3+^ availability. In the absence of Al^3+^, the tea plants failed to generate new roots, and the root tips were damaged within 1 d of Al deprivation. Structural analysis of root tips demonstrated that Al was required for root meristem development and activity. *In situ* morin staining of Al^3+^ in roots revealed that Al mainly localized to nuclei in root meristem cells, but then gradually moved to the cytosol when Al^3+^ was subsequently withdrawn. This movement of Al^3+^ from nuclei to cytosols was accompanied by exacerbated DNA damage, which suggests that the nuclear‐targeted Al primarily acts to maintain DNA integrity. Taken together, these results provide novel evidence that Al^3+^ is essential for root growth in tea plants through maintenance of DNA integrity in meristematic cells.

## Graphical abstract

Ionic aluminium (Al^3+^) has been known to be rhizotoxic to most plant species. However, we demonstrate that Al^3+^ is essential for tea root growth and development through maintenance of DNA integrity in meristematic cells.

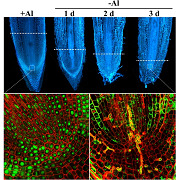

## INTRODUCTION

Aluminium (Al) is the most abundant metal and third most abundant element in the Earth's crust. As soil pH decreases, the solubilization of Al from rock ore increases, which typically leads to the accumulation of soluble ionic Al (Al^3+^) in highly acidic soils (Kinraide 1991). Toxicity of Al^3+^ to most plants is readily apparent, as exposure to micromolar concentrations of Al^3+^ rapidly leads to root damage and significant inhibition of root growth, and the associated reduction in water and mineral nutrient absorption results in sizable yield losses (Kochian 1995; Ma 2007; Ryan et al. 2011).

The initial target of Al toxicity is root tips (Ma 2007), where Al binding sites are mainly localized to the cell walls of the root meristem, elongation zone and the distal portion of the transition zone between the regions of cell division and elongation (Doncheva et al. 2005). The ensuing rapid and strong binding may reduce cell wall extensibility (Yang et al. 2008; Li et al. 2020), and can disrupt plasma membrane surface electrical potentials, altering mineral ion activities at the outer face of the plasma membrane (Kinraide 1991). Many researchers have reported that both external detoxification and internal sequestration contribute to plant resistance to Al toxicity (Ma et al. 2001; Liu et al. 2014). External detoxification involves secretion of organic acid anions, phenolic compounds, and polysaccharides in root mucilage that all form nontoxic Al complexes (Li et al. 2000; Kochian et al. 2004; Ma 2007), increase of rhizosphere pH which reduces Al^3+^ availability (Degenhardt et al. 1998), and decreased capacity of cell walls to bind Al^3+^ (Yang et al. 2008, 2011). Internally, upon entry into the cytoplasm, Al may be complexed by organic acids and phosphate anions, and then sequestrated into vacuoles as chelated Al compounds (Kochian 1995; Ma et al. 1997), which results in high cellular Al content, but with little damage to other cytoplasmic structures and organelles (Ma et al. 2001; Shen and Ma 2001; Shen et al. 2002).

Besides these well documented phytotoxicity and resistance responses, appropriate concentrations of Al^3+^ can also stimulate the growth of specific plant species, especially those native to acidic soils (Pilon‐Smits et al. 2009), including *Melastoma malabathricum* (Watanabe et al. 2005), *Quercus serrata* (Tomioka et al. 2005), *Symplocos paniculata* (Schmitt et al. 2016), and *Eucalyptus gummifera* (Mullette 1975). These plants are all adapted to acidic soils and can accumulate high concentrations of Al inside their cells on acid soils. For example, Al addition to hydroponic nutrient solution at low pH (pH 4) promoted root elongation in *Melastoma malabathricum*, an Al hyperaccumulater with leaf Al concentrations as high as 7 000 mg of Al per kg dry weight (Watanabe et al. 2005). The beneficial effects of Al have been speculated to be due to elevated uptake of essential nutrients (e.g., phosphate), or alleviation of effects associated with other detrimental factors (e.g., Fe^2+^ and/or H^+^ toxicity) (Osaki et al. 1997; Watanabe et al. 2006; Chen et al. 2011). However, a mechanistic basis for Al‐promoted plant growth have yet to be elucidated.

Tea (*Camellia sinensis*), a typical Al hyperaccumulator, is distributed in tropical and subtropical areas of the world where soils are mainly acidic (Matsumoto et al. 1976; Cuenca et al. 1990). Adaptation of tea plants to acidic soils is unlikely due to its high tolerance of high Al concentrations, but rather likely results from a preference for Al in acid soils (Konishi et al. 1985). Tea roots have been shown to exhibit greater root growth in the presence of Al (Fung et al. 2008; Mukhopadyay et al. 2012). In tea shoots, Al is also capable of stimulating pollen tube growth, bud emergence, and increasing leaf photosynthesis and metabolism (Chen et al. 2011; Mukhopadyay et al. 2012). High concentrations of Al in tea shoots (up to 3% of dry matter) may exist in the form of Al‐oxalic acid complexes (Morita et al. 2008, 2011). On the whole, these results suggest that Al plays beneficial roles in tea plant growth and development, although specific information on how Al is involved in these responses remains scarce.

In this study, we demonstrated that Al^3+^ is essential for root growth and development in tea plants. In the absence of Al^3+^, cell differentiation in root meristems rapidly diminished, which quickly resulted in cessation of root growth. Furthermore, this resulted in a significant decrease in nuclear Al content, while nuclear DNA damage increased remarkably at the same time in root meristem cells. These results demonstrate that Al is essential for root growth in tea plants mainly through maintenance of DNA integrity in root meristem cells, during growth in acidic media.

## RESULTS

### Aluminium promotes new root growth in tea plants

**Figure 1:**
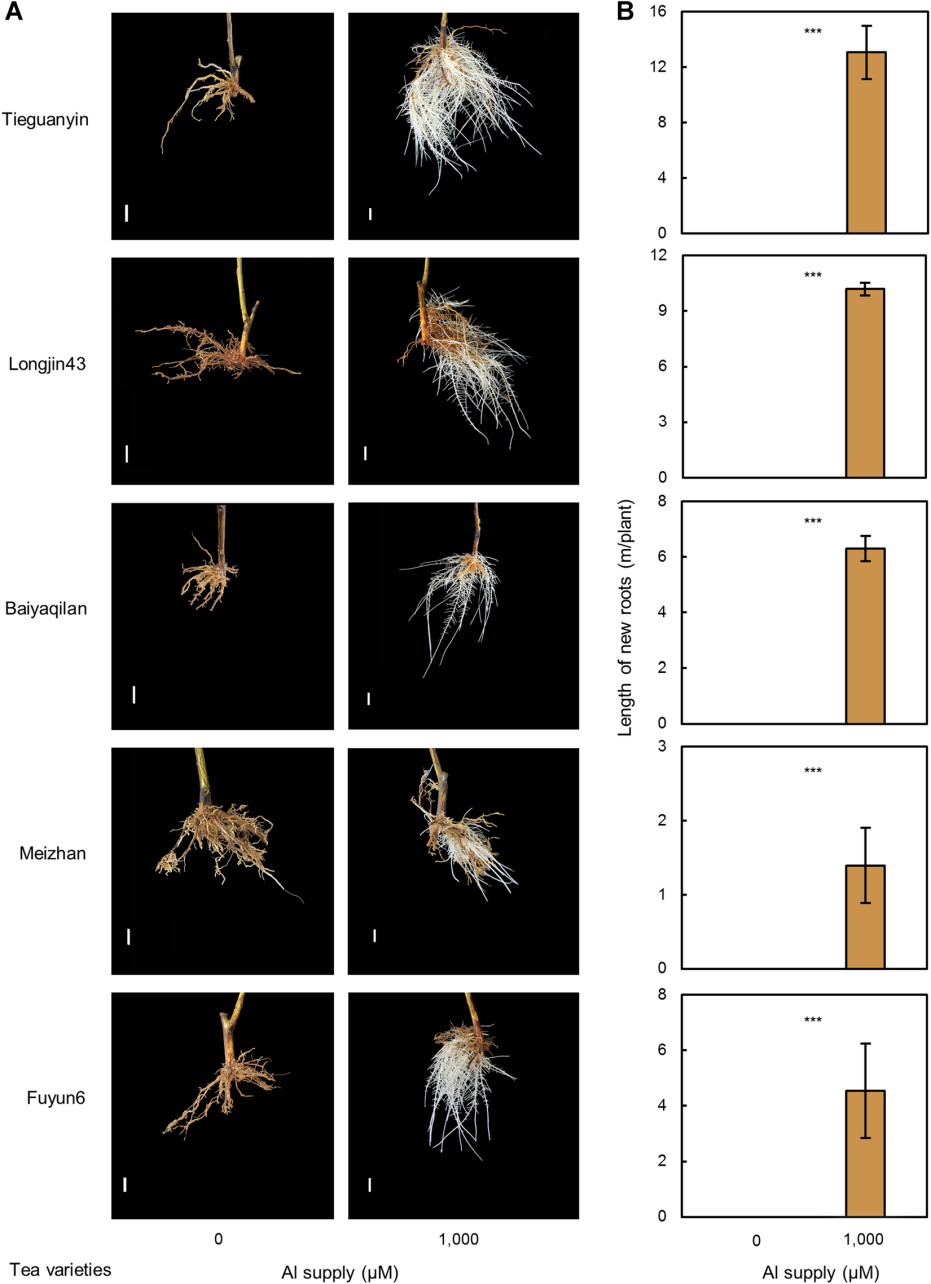
**Effects of aluminium (Al) on root growth of five tea varieties** The cuttings from 1‐year‐old uniform seedlings of five tea varieties Tieguanyin, Longjin43, Baiyaqilan, Meizhan, and Fuyun6 were harvested from the field acid soil and washed in water for 3 d to remove adhering soil particles, and then transferred to hydroponic nutrient solution containing 0 (−Al) or 1,000 (+Al) µmol/L of added Al, at pH 4.5, and grown for 30 d. (**A**) Photographs of root growth phenotypes. Bars = 10 mm. (**B**) Length of new roots under +/−Al growth conditions. Data are means with standard error (*n* = 3). Asterisks indicate significant differences between treatments in *t*‐tests. ***P < 0.001.

To investigate the physiological roles of Al in tea root growth, five popular tea varieties, Tieguanyin, Longjin43, Baiyaqilan, Meizhan, and Fuyun6, were first pregrown in deionized water for 3 d to remove residual Al from root surfaces, and then grown for 30 d in nutrient solution containing 1,000 µmol/L or 0 µmol/L AlCl_3_ (Figure 1). Interestingly, external addition of Al^3+^ did not inhibit root growth, but rather stimulated new root generation and elongation in all of the tested tea varieties, demonstrating that Al does indeed promote tea root growth at acidic pH values. Moreover, in the absence of added Al^3+^ (−Al), no further growth was observed for old roots and no new roots emerged from existing roots in any of these five varieties of tea (Figure 1A, B). These results suggest that Al is essential for tea root growth.

**Figure 2:**
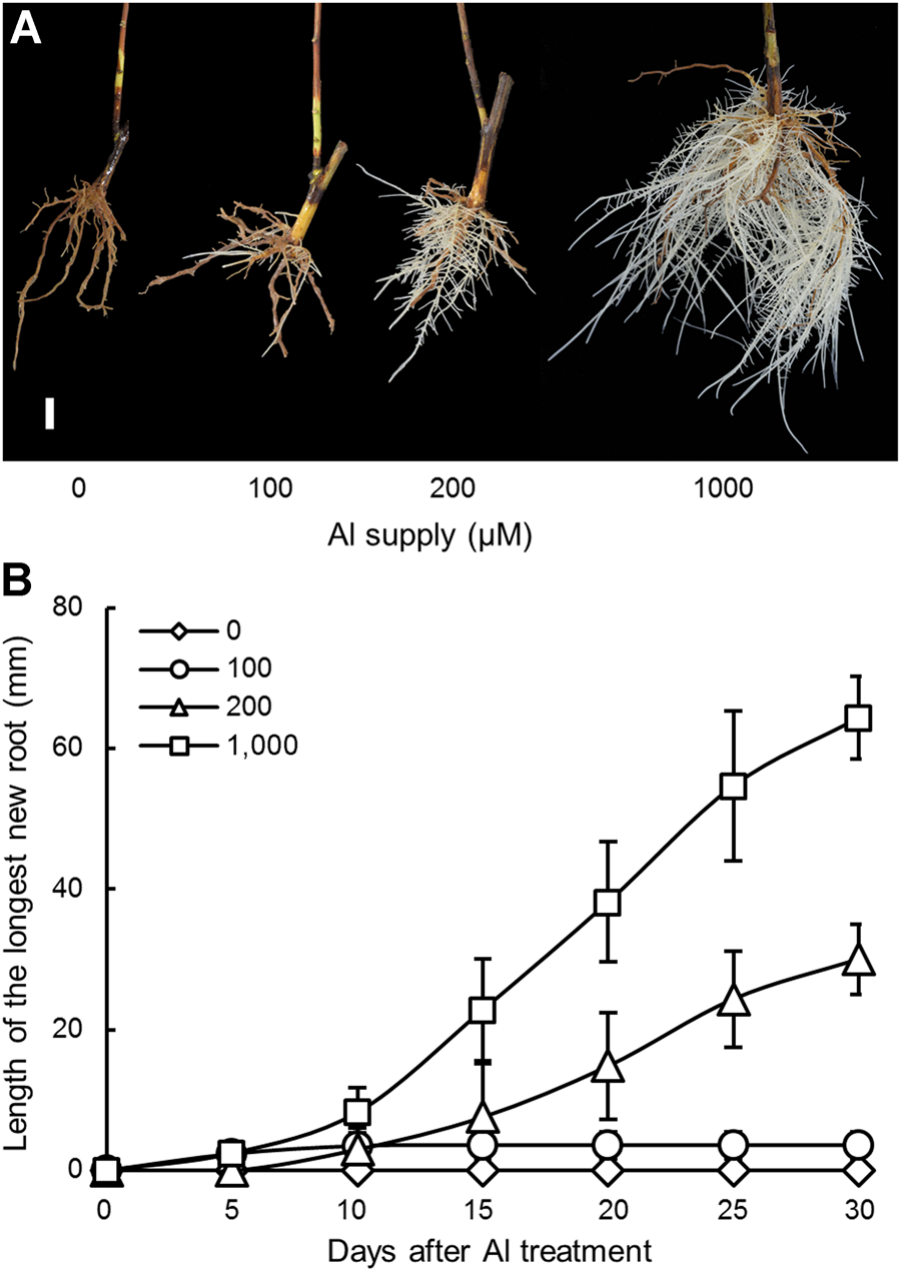
**Effects of varying aluminium (Al) concentrations on Tieguanyin tea root growth** Tieguanyin tea plants were harvested from field acid soil and then transferred to nutrient solution as described in the legend for Figure 1, and then grown for 30 d in nutrient solution containing 0, 100, 200, or 1,000 µmol/L AlCl_3_, at pH 4.5. (**A**) Photographs of root growth phenotypes. Bar = 10 mm. (**B**) Length of the longest new root. Data are means with standard error (*n* = 5).

One of the tested tea varieties, Tieguanyin, was then used for further detailed analysis of Al^3+^ concentration and time‐dependent effects on tea root growth in nutrient solution at pH 4.5, which has been reported as the optimal pH for tea plant growth (Konishi et al. 1985). In addition, Al^3+^ activity in nutrient solutions was determined in order to assess the availability of the rhizotoxic form of Al, Al^3+^ ions (Figure S1). Almost no new roots emerged in the 0 and 100 µmol/L (Al^3+^ activity: 47.1 µmol/L) Al treatments, while new roots started to emerge at d 10 and kept growing in the 200 (Al^3+^ activity: 119.5 µmol/L) and 1,000 µmol/L (Al^3+^ activity: 195.7 µmol/L) Al supply treatments (Figure 2A, B). Furthermore, the length of new roots in 1,000 µmol/L Al was twice that of new roots in 200 µmol/L Al (Figure 2B). This showed that Al promotion of tea root growth acts in a dose‐dependent manner between Al^3+^ activities of around 120–200 µmol/L.

### Promotion of new root growth is highly dependent on Al^3+^ availability and not proton toxicity

**Figure 3:**
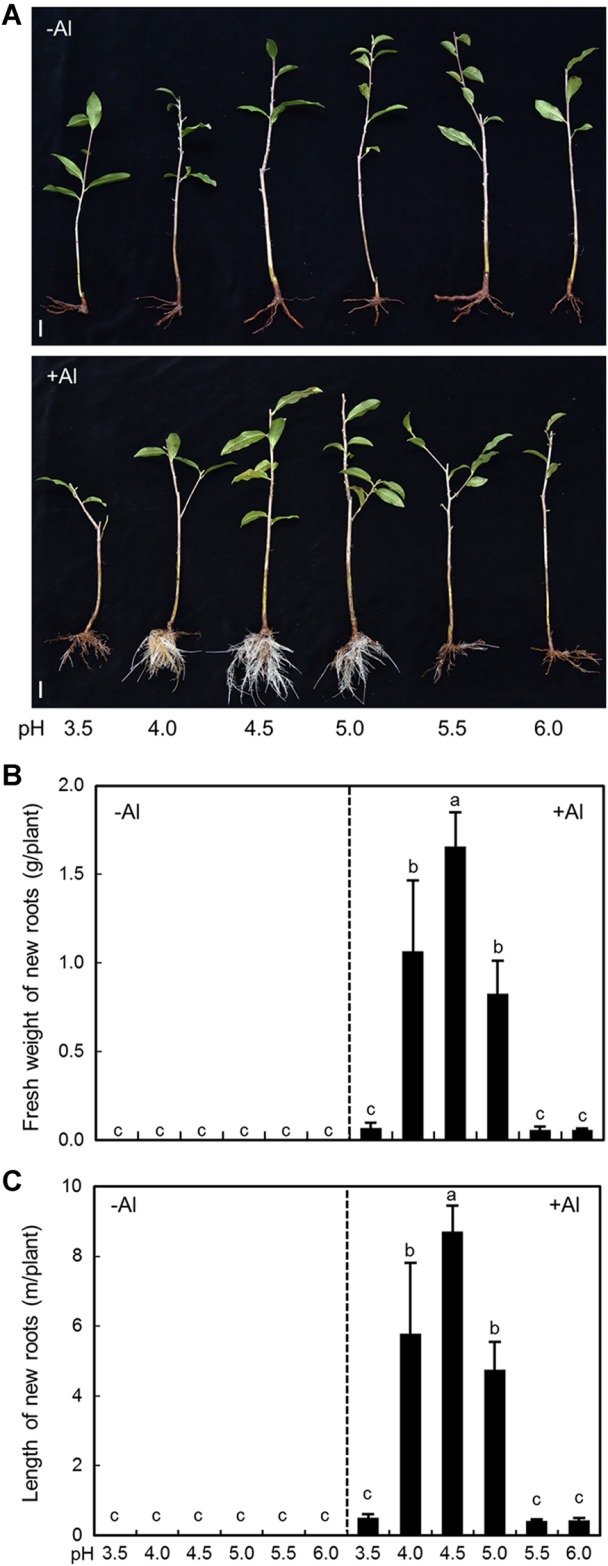
**Effects of pH on Tieguanyin root growth** Tieguanyin tea plants were harvested from field acid soil and then transferred to nutrient solution as described in the legend for Figure 1, and then grown for 30 d in nutrient solution containing 0 or 400 µmol/L AlCl_3_ at pH values of 3.5, 4.0, 4.5, 5.0, 5.5, or 6.0. (**A**) Photographs of plant growth phenotypes. Bars = 20 mm. (**B**) Fresh weight of new roots. (**C**) Length of new roots. Data are means with standard error (*n* = 3). Different letters indicate significant differences between treatments (*P* < 0.05) in Duncan's multiple range test.

Given that pH has large effects on the availability of Al species, and in particular Al^3+^ at pH values below 5.5 (Kinraide and Parker 1989), we further investigated the combined effects of pH and Al on tea root growth. In the absence of added Al, tea roots failed to grow, regardless of pH values ranging from 3.5 to 6 in the nutrient solution (Figure 3A–C). On the other hand, in the presence of 400 µmol/L AlCl_3_, the fresh weight and length of new roots increased significantly, depending on the nutrient solution pH values (Figure 3B, C). The greatest root biomass and length increases were observed at a nutrient solution pH of 4.5 (Figure 3A–C). As pH increased from 4.5 to 6, root growth declined, possibly due to the reduced availability of ionic Al^3+^ with increasing pH. Toward the other extreme, as pH dropped from 4.5 to 4.0 and 3.5, root growth also declined, suggesting that tea roots might have suffered from proton toxicity under these extremely low pH conditions (Figure 3A–C). These results indicate that the promotion of root growth by Al are reversed when the nutrient solution pH became highly acidic (pH <4), due to proton toxicity effects on new root growth.

### Aluminium is essential for tea root growth and development, and is required to maintain root tip meristem activity

**Figure 4:**
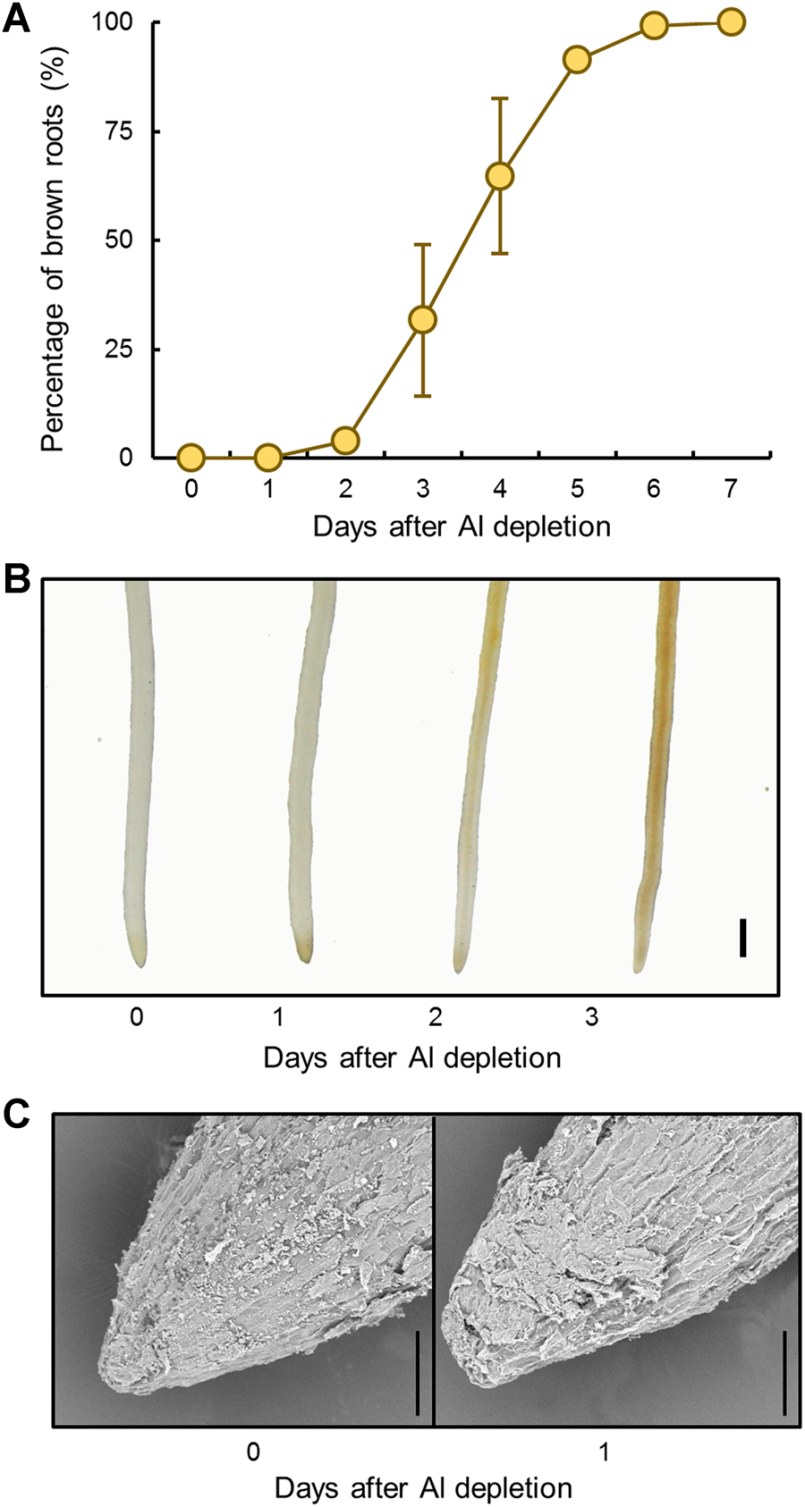
**Effects of aluminium (Al) depletion on root tip morphology for Tieguanyin roots over a 7 d period of ‒Al treatment** After 30 d of growth in nutrient solution containing 400 µmol/L Al at pH 4.5, Tieguanyin tea plants were transferred to solution lacking added Al for an additional 7 d. (**A**) Percentage of brown roots relative to the total number of roots over time. Data are means with standard error (*n* = 12). (**B**) Photographs of roots showing development of brown discoloration. Bar = 1 mm. (**C**) Photographs of root tip surface imaged using scanning electron microscopy. Bars = 100 µm.

To further clarify whether Al is essential or beneficial for tea root growth and development, tea plants were first precultured in acidic nutrient solution with Al for 30 d in order to obtain new healthy white roots, which was then followed by transplanting into 0 or 400 µmol/L AlCl_3_ nutrient solution treatments for another 7 d (Figure 4). Within just 2 d of removal of supplied Al, healthy white roots turned brown, and, by d 6 of withholding Al, the roots died (Figure 4A, B). Closer microscopic observation revealed that after 1 d of Al deprivation, root tips were remarkably damaged, as reflected by changes in the shape of root tips from conical to flattened in scanning electron micrographs (Figure 4C). These results demonstrated that Al is essential for development of healthy roots in tea plants when grown at pH 4.5.

**Figure 5:**
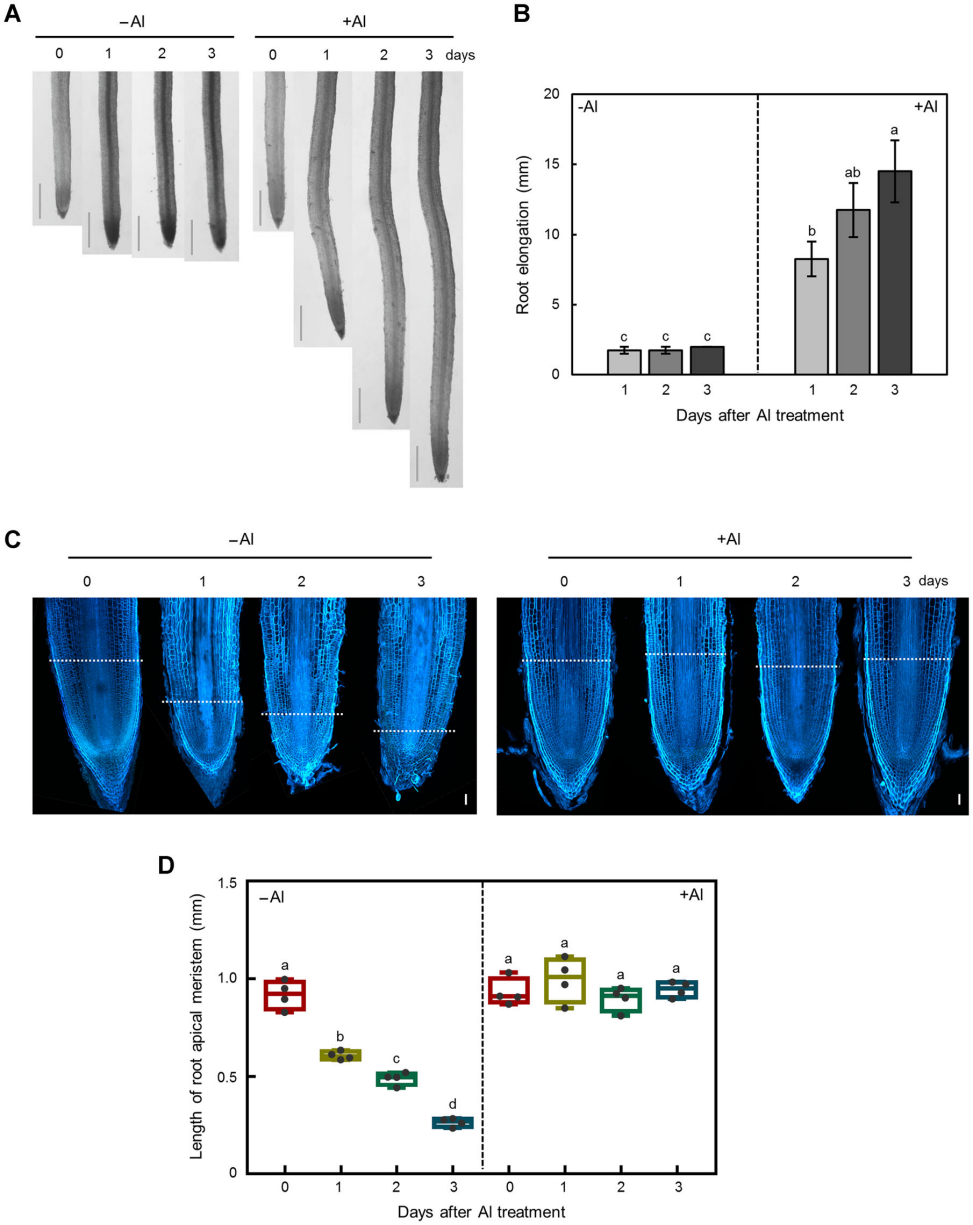
**Effects of 3 d +/−Al treatment on root development and elongation after growth on +Al solution for 30 d** After 30 d of growth in nutrient solution containing 400 µmol/L Al at pH 4.5, Tieguanyin tea plants were transferred to solution containing 0 or 400 µmol/L AlCl_3_ for an additional 3 d. (**A**) Photographs of root phenotypes. Bars = 2 mm. (**B**) Root elongation. Data are means with standard error (*n* = 4). (**C**) Photographs of root tip longitudinal sections. The blue color indicates the fluorescence signal of Calcofluor‐white bound to root cell walls. Bars = 100 µm. (**D**) Length of meristem zone. Data are means with standard error (*n* = 4). Different letters indicate significant differences between treatments (*P* < 0.05) in Duncan's multiple range test.

Roots that were kept in 400 µmol/L AlCl_3_ kept elongating, whereas roots transferred to nutrient solution without any additional Al supplied ceased to elongate (Figure 5A, B). Calcofluor‐white staining of the root apical meristem zones revealed that the length of the root meristematic zones in roots of tea plants subjected to Al deprivation decreased over time to only 61.0% (d 1), 54.5% (d 2) and 27.9% (d 3) of the lengths observed in roots supplied with 400 µmol/L AlCl_3_ (Figure 5C, D). This strongly indicated that Al is important for maintenance of the meristematic zone in emerging and growing tea root tips.

**Figure 6:**
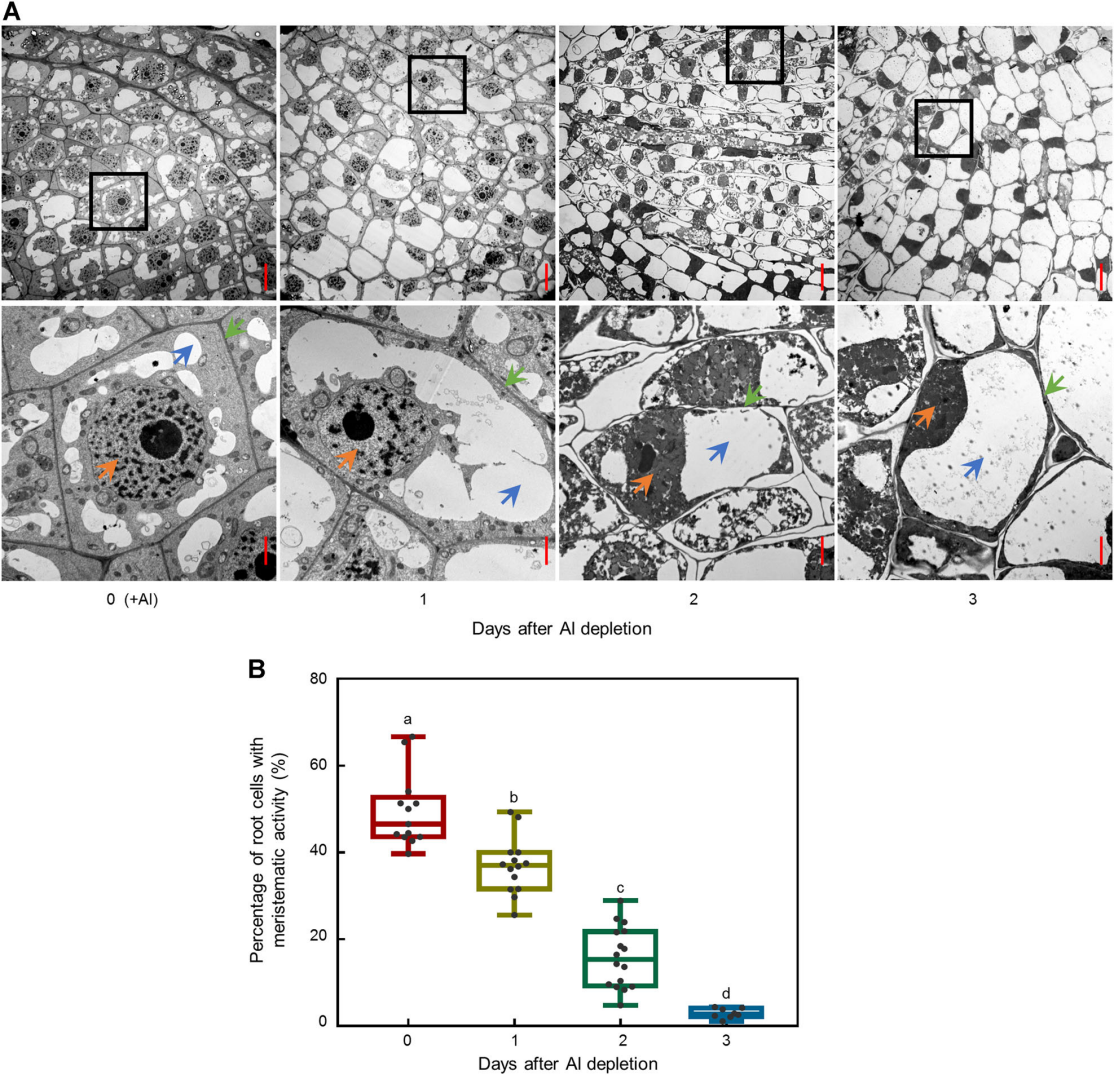
**Effects of aluminium (Al) on cell ultrastructure in tea root apices** After growth in nutrient solution supplemented with added Al for 30 d, tea plants were transferred into nutrient solution lacking Al for an additional 3 d. (**A**) Transmission electron micrographs of root tip cellular ultrastructure. Images in the lower row are enlargements of the image area in black boxes in the upper row of micrographs. Orange, blue, and green arrows denote the nucleus, vacuole, and cell wall, respectively. Bars = 10 µm (upper line) and 2 µm (lower line). (**B**) Percentage of root cells with meristematic activity. Cells with larger nuclei, scattered vacuoles and thin cell walls were regarded as meristematically active. Data are means with standard error (*n* = 13, 14, 16, and 8 for 0, 1, 2, and 3 d, respectively). Different letters indicate significant differences between treatments (*P* < 0.05) in Duncan's multiple range test.

Further examination of the effects of Al on the ultrastructure of meristematic cells was conducted with root tip samples harvested from tea plants exposed to nutrient solution containing 0 or 400 µmol/L AlCl_3_ for 1, 2, or 3 d using transmission electron microscopy. When Al was provided in the nutrient solution, root meristematic cells were relatively small and compact, and contained large and prominent nuclei in their cell centers, with small vacuoles surrounding the nucleus (Figure 6A). When Al was withheld from the nutrient solution, cells in the meristematic zones stopped dividing and were surrounded by thickened cell walls (Figure 6A). Moreover, the nuclei of these cells were smaller than those in roots harvested from nutrient solution supplied with Al, while the vacuoles increased in size and eventually pushed the nuclei away from the center of the cells (Figure 6A). Accordingly, the percentage of cells exhibiting meristematic activity (characterized by large nuclei, scattered vacuoles and thin cell walls) decreased dramatically with prolonged of Al depletion, suggesting that Al fills essential roles in the maintenance of root meristematic activity in tea plants.

Taken together, these results clearly demonstrate that Al is required to maintain meristematic activity in the root tips of tea plants.

### Nuclear‐targeted Al is essential to maintain the integrity of DNA in the root tips of tea plants when grown in low pH media

**Figure 7:**
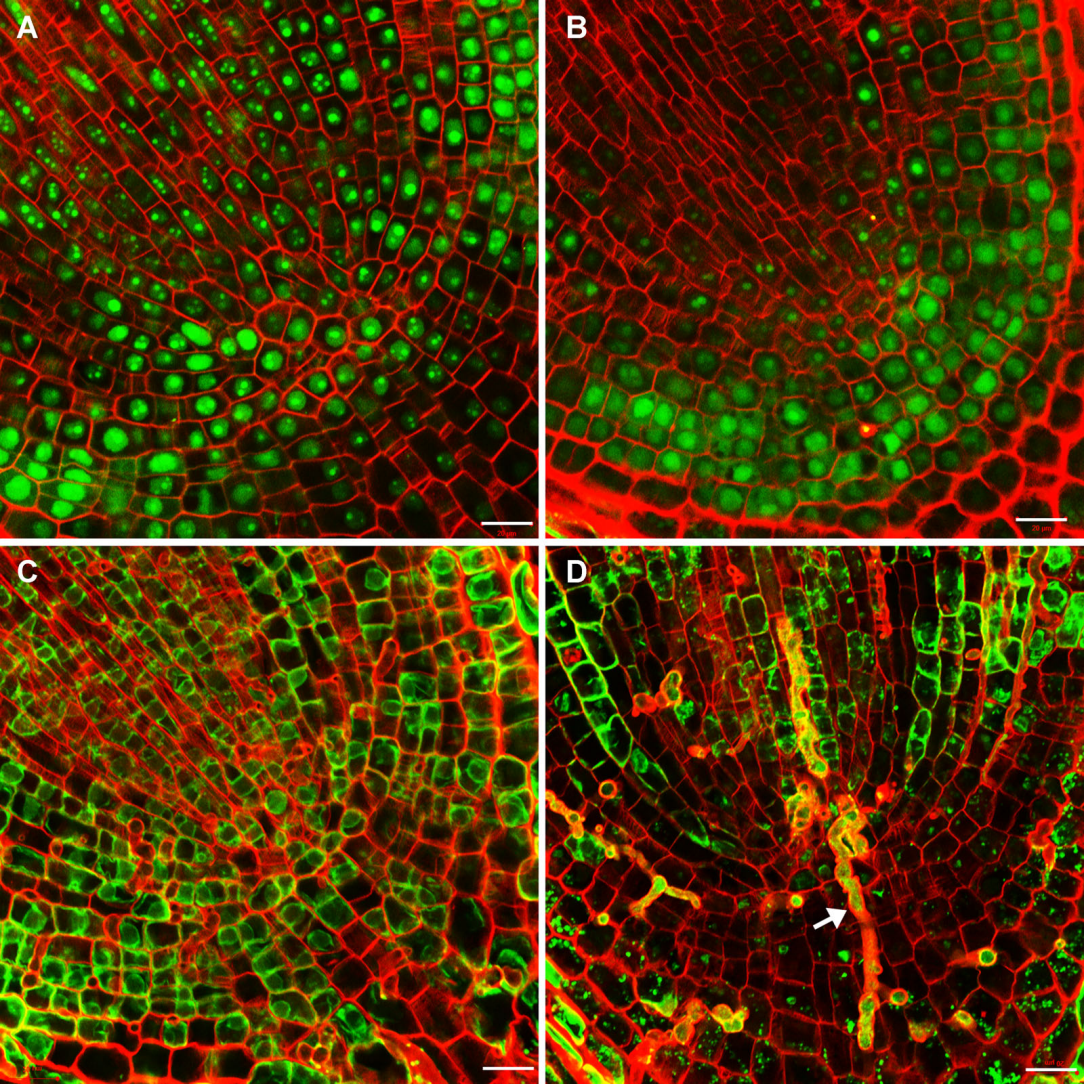
**Localization of aluminium (Al) in tea plant root tips** After growth in nutrient solution supplemented with Al for 30 d, tea plants were transferred to nutrient solution lacking added Al for an additional 3 d. Root cell structures were imaged after withdrawal of Al for 0 (**A**), 1 (**B**), 2 (**C**), and 3 (**D**) d. The green fluorescence is due to the morin staining of Al in the root tip cells, while the Calcofluor‐white staining shown in red shows the structure of root cell walls. The white arrow points to abnormal cells. Bars = 20 µm.

*In situ* morin staining was carried out to investigate the distribution of Al within root cells. Consistent with previous findings (Hajiboland and Poschenrieder 2015), the morin signal in cross‐sections of the root meristem (2 mm from root apex) were mainly distributed in the cell wall under relatively low Al (100 µmol/L) conditions (Figure S2). With sufficient Al (1,000 µmol/L) continually supplied in nutrient solution, a strong morin signal was observed mainly in the nuclei of root meristem cells, indicating the Al was accumulating in the nuclei of cells in actively growing roots (Figures 7A, S2). Over the first 2 d of Al deprivation, the nuclear morin signals weakened significantly, while cytoplasm morin signal intensified (Figure 7B, C). After 3 d of Al deprivation, the morin signal was nearly nonexistent in meristematic cells, which was accompanied by remarkable cell morphological changes, as indicated by increases in the prevalence of abnormal cells (Figure 7D).

**Figure 8:**
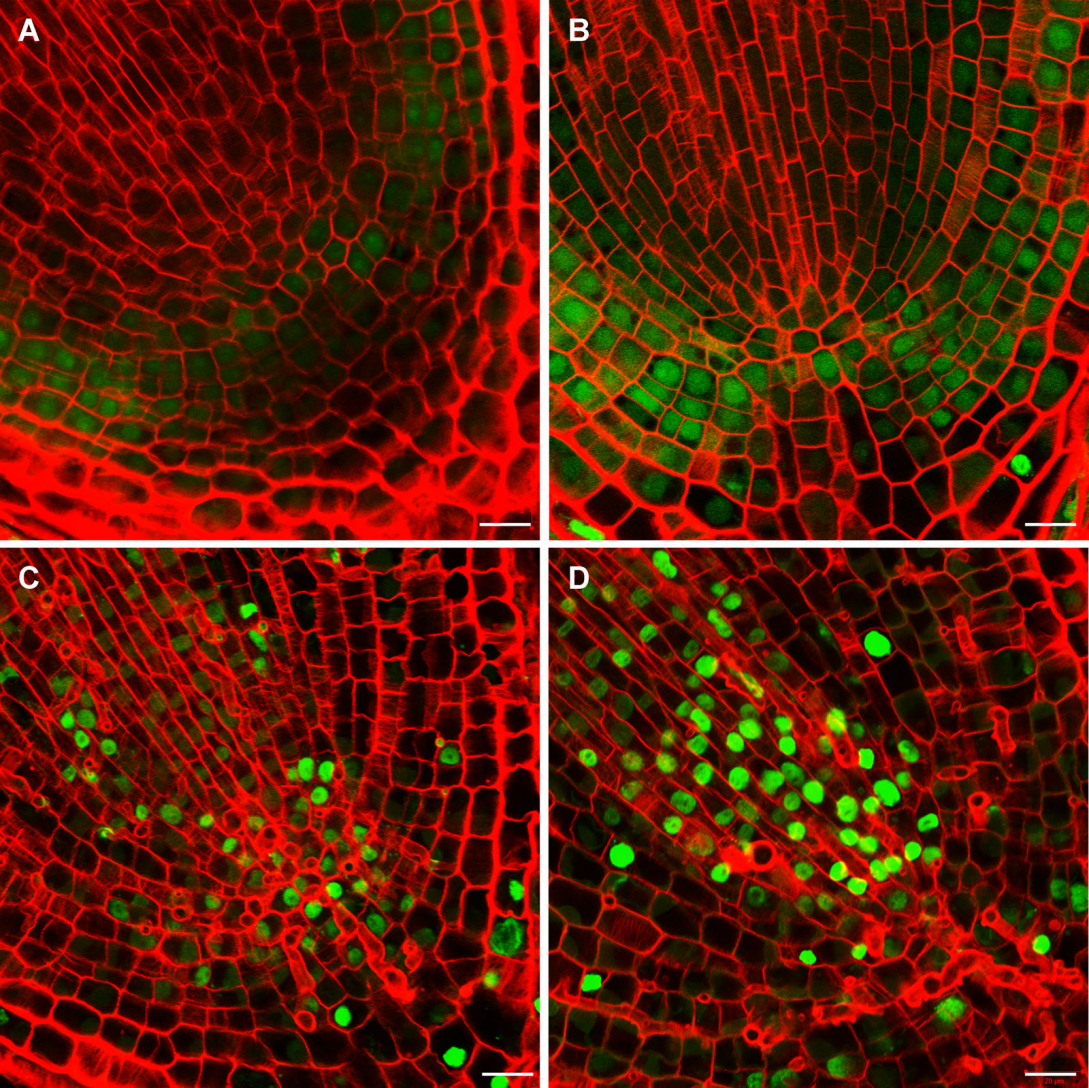
**DNA damage in tea plant root tips** After growth in nutrient solution supplemented with aluminium (Al) for 30 d, tea plants were transferred into nutrient solution lacking added Al for an additional 3 d. Root cell structures were imaged after withdrawal of Al for 0 (**A**), 1 (**B**), 2 (**C**), and 3 (**D**) d. Terminal deoxynucleotidyl transferase dUTP nick end labeling (TUNEL) staining due to DNA fragmentation and damage is indicated by the green staining, while Calcofluor‐white staining shown in red depicts the root cell walls. Bars = 20 µm.

To gain more insight into what is occurring in nuclei of Al‐deprived root meristematic cells, terminal deoxynucleotidyl transferase dUTP nick end labeling (TUNEL) assays were performed to assess DNA integrity. Compared to the cells observed at the onset of Al deprivation (0 d; Figure 8A), the green nuclear TUNEL signals in meristematic cells were enhanced and increased with increasing time in Al deprivation conditions (Figure 8B–D). This suggests that the frequency of nuclear DNA breaks increased in response to Al depletion. Taken in conjunction with the other results presented herein, it appears that Al is mainly localized to the nuclei of tea root meristematic cells, where it plays currently unexplained fundamental roles in maintaining DNA integrity when roots are grown in conditions that mimic acid soils, and thereby assists in the growth and development of root tips, root elongation, and healthy tea plants.

## DISCUSSION

For most plant species, Al^3+^ in acid soils stunts root growth through rapid binding with the cell wall and plasma membrane, resulting in structural and functional changes leading to damaged root systems. Also, Al toxicity is associated with production of reactive oxygen species, which also presumably result in cellular damage. These pleiotropic effects of Al toxicity result in a diminished root system that leads to greatly reduced root water and nutrient uptake, and crop yield reduction (Ma 2000; Kochian et al. 2004). However, Al also has been suggested to have beneficial effects for some plant species that are well adapted to growth on highly acidic soils (Pilon‐Smits et al. 2009; Metali et al. 2012). The beneficial effects of Al on tea plant growth has been documented for decades; however, the underlying mechanisms had not been investigated in any detail (Chenery 1955; Konishi et al. 1985; Yokotal et al. 1997; Mukhopadyay et al. 2012).

In this study, we first investigated the physiology effects of Al on root growth and elongation in five different tea varieties, to provide solid evidence that Al does indeed promote tea root growth under acidic growth conditions (Figure 1). The beneficial role of Al likely involves common and conserved mechanisms among tea varieties, as all of the varieties observed in this study generated considerable numbers of new roots with exposure to Al in the pH 4.5 nutrient solution (Figure 1). Furthermore, this stimulation was highly dependent on Al concentration in the nutrient solution (Figure 2), with new root growth improving significantly with increasing Al availability from 100 to 1,000 μmol/L (Figure 2A, B), which are usually extremely phytotoxic levels to most plant species. These results suggest that, as in previous studies (Konishi et al. 1985), the adaptation of tea plants to high Al conditions is more likely due to the preference for Al, rather than a high resistance to toxic effects of Al.

Further experimentation revealed that Al is not just beneficial, but actually is essential for tea root growth and development. Evidence supporting this conclusion comes from our observations that the established roots from tea plants transplanted from acid soils to hydroponics stopped growing and no new roots emerged when tea plants were incubated in complete nutrient solution without any additional Al being supplied (Figures 1–3). In contrast, healthy white roots did emerge and kept growing when Al was added to nutrient solution (Figures 1–3). Moreover, when plants were transferred from nutrient solution containing Al to Al‐deficient solution, new roots quickly stopped growing and turned brown before losing vitality and dying (Figure 4). These results meet the first criterion for an element to be considered an essential plant nutrient in that the plant is unable to complete its life cycle in the absence of the element (Arnon and Stout 1939). In addition, given that the nutrient solution provided in this study contains all of the mineral elements considered essential for plants to date, none of the other included 14 mineral elements can functionally substitute for Al in tea roots. Aluminium, therefore, meets another criterion for being considered an essential nutrient element, in that the function of this element is irreplaceable by any other mineral element (Arnon and Stout 1939). Furthermore, our results demonstrate that Al is crucial for maintenance of DNA integrity in meristematic cells, which is required for cell division and subsequent root elongation (Figures 5–8). This meets the third criterion for being considered an essential nutrient element in that this element must be directly involved in plant metabolism, although Al functions at physiological or molecular levels need to be further investigated. Based on these results, it is unavoidable to conclude that Al is an essential element for tea plant growth, rather than a beneficial element.

It has been proposed that Al may alleviate H^+^ toxicity effects in low pH conditions for some plants exhibiting beneficial responses to Al (Osaki et al. 1997). However, the pH gradients and Al combinations included in this study demonstrated that the requirement of Al is not indirectly caused by alleviation of H^+^ toxicity, as elevating the nutrient solution pH to 6.0 in the Al deprivation treatments did not lead to improvements in root growth (Figure 3). Instead, when compared with what we observed were optimal growth conditions (pH 4.5, Al present), elevating the pH from 4.5 to 6.0 led to worse root growth in the presence of Al (Figure 3). This suggests that ionic Al^3+^, but not H^+^ activities, may contribute to tea root growth. Other researchers have uncovered molecular evidence that plants are capable of actively acquiring Al^3+^ (Xia et al. 2010; Wang et al. 2017). Tea plants might have developed similar transport systems for Al uptake. Our study does provide evidence that ionic Al^3+^ accumulates within the nucleus of root meristem cells, where it is important for the maintenance of DNA integrity. Upon withdrawal of supplied Al, nuclear Al quickly moved out of the nucleus to the cytoplasm (Figure 7B–D), and this alteration in Al cellular localization correlated closely with the time course for DNA damage becoming prominent in meristematic cells (Figure 8B–D), which resulted in the significant loss of meristematic activity (Figure 6) and loss of root growth (Figures 4, 5). However, it could not rule out a possibility that the maintenance of DNA integrity is a result of Al‐dependent tea root growth.

Aluminium binding sites in DNA within tea root tip cells might be the phosphodiester backbone, where negative charges exist (Karlik et al. 1980). In *Arabidopsis*, strong binding of Al to DNA triggers ATR‐, ALT2‐, and SOG1‐regulated transcriptional responses which actively halt root growth (Sjogren et al. 2015). Despite the dramatic physiological differences between *Arabidopsis* and tea plants in response to Al exposure, the findings that Al binding to root cell DNA in *Arabidopsis* triggers transcriptional responses linked to root growth allows us to speculate that in tea, nuclear localized Al may be involved in stabilizing DNA and mitosis through regulation of related genes. However, the molecular mechanisms underlying the active responses of tea roots to Al exposure are well beyond the scope of this study and will certainly be the subject of future investigations.

The adaptation of tea plants to acidic soils was long thought to be due to their expression of high resistance to Al toxicity via secretion of organic acids to externally detoxify Al and also due to Al sequestration internally (Morita et al. 2004, 2008, 2011), along with concomitant increases in the uptake of essential nutrients (e.g., Ca, Mg, K) and alleviation of toxic effects of other elements (e.g., F, Fe) (Fung et al. 2008; Hajiboland et al. 2013; Yang et al. 2016). In this study, we present evidence for the novel hypothesis that available Al^3+^ is essential for root growth and development in tea plants through maintenance of DNA integrity and root meristematic activity.

## MATERIALS AND METHODS

### Plant materials and growth conditions

Research was conducted with cuttings from 1‐year‐old uniform seedlings of tea (*Camellia sinensis*) harvested from the field (Anxi County, Fujian, China). The varieties tested included Tieguanyin, Longjin43, Baiyaqilan, Meizhan, and Fuyun6. Cuttings were first precultured in deionized water for 3 d to remove adhering soil particles. Seedlings were then grown for 1 month (30 d) in nutrient solution containing either 0 or 1,000 µmol/L AlCl_3_, at a pH of 4.5. The nutrient solution contained 250.0 μmol/L (NH_4_)_2_SO_4_, 62.5 μmol/L Ca(NO_3_)_2_, 37.5 μmol/L CaCl_2_, 25.3 μmol/L KH_2_PO_4_, 125.0 μmol/L K_2_SO_4_, 100.0 μmol/L MgSO_4_, 16.4 μmol/L Fe(III)‐ethylenediaminetetraacetic acid, and small quantities of H_3_BO_3_, MnSO_4_, ZnSO_4_, CuSO_4_, and (NH4)_6_Mo_7_O_24_. The root system of each seedling was photographed using a digital camera (D610, Nikon, Japan). Additionally, the length of new roots was measured after 1 month of growth in +/− Al treatments using WinRHIZO (LA2400; Regent, Canada), with root images captured by a scanner (1640XL; Epson, Japan).

In addition, Tieguanyin seedlings were also studied for 1 month in an Al dosage‐response experiment that included tea plants grown in nutrient solution supplemented with 0, 100, 200, or 1,000 µmol/L of AlCl_3_ at pH 4.5. The length of the longest new root on each plant was measured by ruler every 5 d over the 30 d growth period for a total of six measurements per root system.

Another experiment was conducted to investigate the effect of pH and Al^3+^ interactions on Tieguanyin tea seedlings grown for 1 month in nutrient solution containing 0 or 400 µmol/L AlCl_3_ with the solution pH adjusted to 3.5, 4.0, 4.5, 5.0, 5.5, or 6.0. New roots were scanned for total length measurements and then root system biomass determined via measurement of root dry weight after drying root systems in a 65 °C oven for 4 d.

To investigate the effect of withdrawing Al from the nutrient solution after an initial growth on Al‐containing nutrient solution, Tieguanyin tea seedlings were first grown in nutrient solution containing 400 µmol/L AlCl_3_ at pH 4.5 for 1 month prior to transferring plants to nutrient solution containing 0 or 400 µmol/L AlCl_3_ for an additional 7 d. Root lengths were measured daily over the 7 d period, and root elongation was calculated as the root length after treatment minus the root length before treatment. Additionally, the number of brown roots and total number of roots were counted daily. The percentage of brown roots was calculated as the number of brown roots/total number of roots × 100. Root tips (1 cm) were also frozen in TBA (Tert butyl alcohol) over night, dried in a freeze drying system (FreeZone 2.5 Liter; Labconco, USA), and then observed for root surface morphological features via scanning electron microscopy (ProX, Phenom, China). Root tips (1 cm) were also sampled every day using laser confocal microscopy for root structure analysis, transmission electronic microscopy for subcellular ultrastructural alterations, and *in situ* morin (for Al) and TUNEL (for DNA fragmentation in root meristem cells) staining as described below.

### Aluminium ion activity assay

The Al^3+^ activity in the nutrient solution was determined according to the Pyrocatechol violet (PCV) method as described by Dougan and Wilson (1974). After the preparation of nutrient solution supplemented with 100, 200, 400, or 1,000 µmol/L of AlCl_3_ at pH 4.5, Al solution were diluted to 3.5 mL, buffered with 1 mL imidazole buffer (1 mol/L, pH 5.6), colored with 0.2 mL PCV solution (0.0375%), and detected spectrophotometrically at a wavelength of 578 nm using an ultraviolet spectrophotometer (UV‐1780; Shimadzu, Japan).

### Root structure analysis

Root tip samples were excised and imbedded in 5% agar before obtaining longitudinal sections (50 μm) using a microtome (RM2235; Leica, Germany). After staining with Calcofluor‐white (1:1,000; Sigma, USA) for 5 min, these sections were observed under a laser scanning confocal microscope (LSM880; Carl Zeiss, Germany). The apical meristem length (the distance between the quiescent center and the starting point of the elongation zone) was determined through measurements made using Image J software (1.43u; National Institutes of Health, USA).

### Transmission electron microscopy

To observe the ultrastructure of root meristem cells, Tieguanyin tea seedlings were first grown in nutrient solution containing 400 µmol/L AlCl_3_ at pH 4.5 for 1 month. A portion of seedlings was transplanted into the –Al nutrient solution daily, and 3 d after the first transfer, root tips of each day's samples were collected, and then fixed with glutaraldehyde (2.5%) for 2 h, and then along with osmium tetroxide (1%) for another 2 h. Samples were then dehydrated via exposure from a 50% to 95% ethanol gradient, with each gradient step being applied for 15 min, which was followed by immersion in absolute ethanol for 20 min. After fixation, samples were then embedded in Spurr's resin. Well prepared resin samples were sliced into 70 nm thick sections using an ultrathin microtome (UC7; Leica, Germany). Ultrathin sections were stained with uranyl acetate and lead citrate, and imaged using a transmission electron microscope (H‐7650; Hitachi, Japan). Cells in the root meristem zone with large nuclei, scattered vacuoles, and thin cell walls were regarded as being meristematically active. The percentage of root cells with meristematic activity in a visual field was calculated as the number of active meristematic cells/the total number of cells × 100.

### Morin and TUNEL staining

Root tip samples were excised and imbedded in 5% agar prior to excising 50 µm sections using a microtome (RM2235; Leica, Germany). Root transverse sections at 2 mm from the root apex were stained with morin solution (100 µmol/L; Sigma, USA) at room temperature for 30 min. Longitudinal root tip sections were exposed to morin solution for 30 min, or to TUNEL solution (to stain for DNA fragmentation) at 37 °C in the dark for 1 h using *In Situ* Cell Death Detection Kits (Roche, Switzerland). Afterward, sections were washed three times with phosphate‐buffered saline (PBS; pH 7.4, 8 mmol/L Na_2_HPO_4_, 2 mM KH_2_PO_4_, 138 mmol/L NaCl, and 2.7 mmol/L KCl) and then stained with Calcofluor‐white (1:1,000; Sigma, USA) for 5 min. The fluorescence signal was observed via laser scanning confocal microscopy (LSM880; Carl Zeiss, Germany) with the excitation wavelengths set to 405 nm for cell wall signals and 488 nm for morin and TUNEL signals.

## STATISTICAL ANALYSIS

All the data were statistically analyzed by two‐way analysis of variance followed by Duncan's multiple range test using SPSS (v16.0; SPSS, USA).

## AUTHOR CONTRIBUTIONS

H.L. and L.S. designed the experiments and wrote the manuscript. L.S., M.Z., X.L., and C.S. performed the experiments and analyzed the data. Q.M. guided the transmission electron microscopy. L.K. gave comments and careful revisions for this paper. All authors read and approved its content.

## Supporting information

Additional Supporting Information may be found online in the supporting information tab for this article: http://onlinelibrary.wiley.com/doi/10.1111/jipb.12942/suppinfo

**Figure S1.** Al3^+^ activity in nutrient solution
Nutrient solution containing 100, 200, 400, and 1,000 µmol/L of added Al at pH 4.5 were prepared separately and determined for Al3^+^ activity as described in the Materials and Methods. Data are means with standard error (*n* = 3).
**Figure S2.** Localization of Al in root apical meristem of tea plant
Tieguanyin tea plants were grown in solution containing 100 or 1,000 µmol/L AlCl3 for 30 d. Root transverse sections at 2 mm from the root apex were stained with 100 µmol/L morin solution for 30 min, and photographed by confocal microscopy. Bars =  50 µm.
