# Supplementary material for: Aluminium is essential for root growth and development of tea plants (Camellia sinensis)
Source: J Integr Plant Biol. 2020 May 15;62(7):984–97. doi: 10.1111/jipb.12942 (PMC7383589; doi:10.1111/jipb.12942)
Supplement: Supplementary file 1 — Figure S1. Al3+ activity in nutrient solution Nutrient solution containing 100, 200, 400, and 1,000 µmol/L of added Al at pH 4.5 were prepared separately and determined for Al3+ activity as described in the Materials and Methods. Data are means with standard error (n = 3). Figure S2. Localization of Al in root apical meristem of tea plant Tieguanyin tea plants were grown in solution containing 100 or 1,000 µmol/L AlCl3 for 30 d. Root transverse sections at 2 mm from the root apex were stained with 100 µmol/L morin solution for 30 min, and photographed by confocal microscopy. Bars = 50 µm. [file JIPB-62-984-s001.docx]

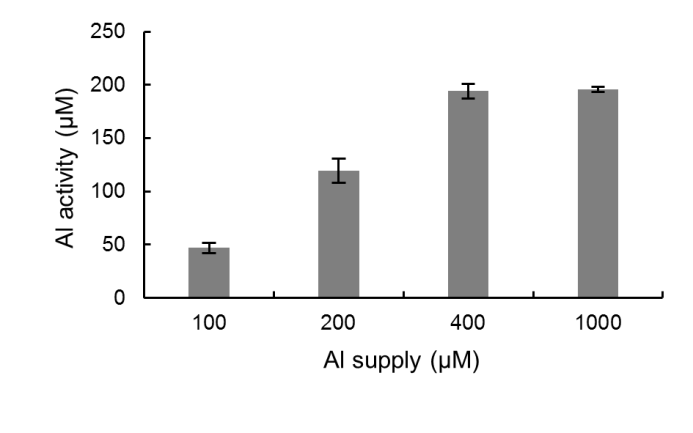


**Supplementary Figure 1. Al^3+^ activity in nutrient solution**

Nutrient solution containing 100, 200, 400 and 1000 µM of added Al at pH 4.5 were prepared separately and determined for Al^3+^ activity as described in the Materials and Methods . Data are means with standard error (n=3).


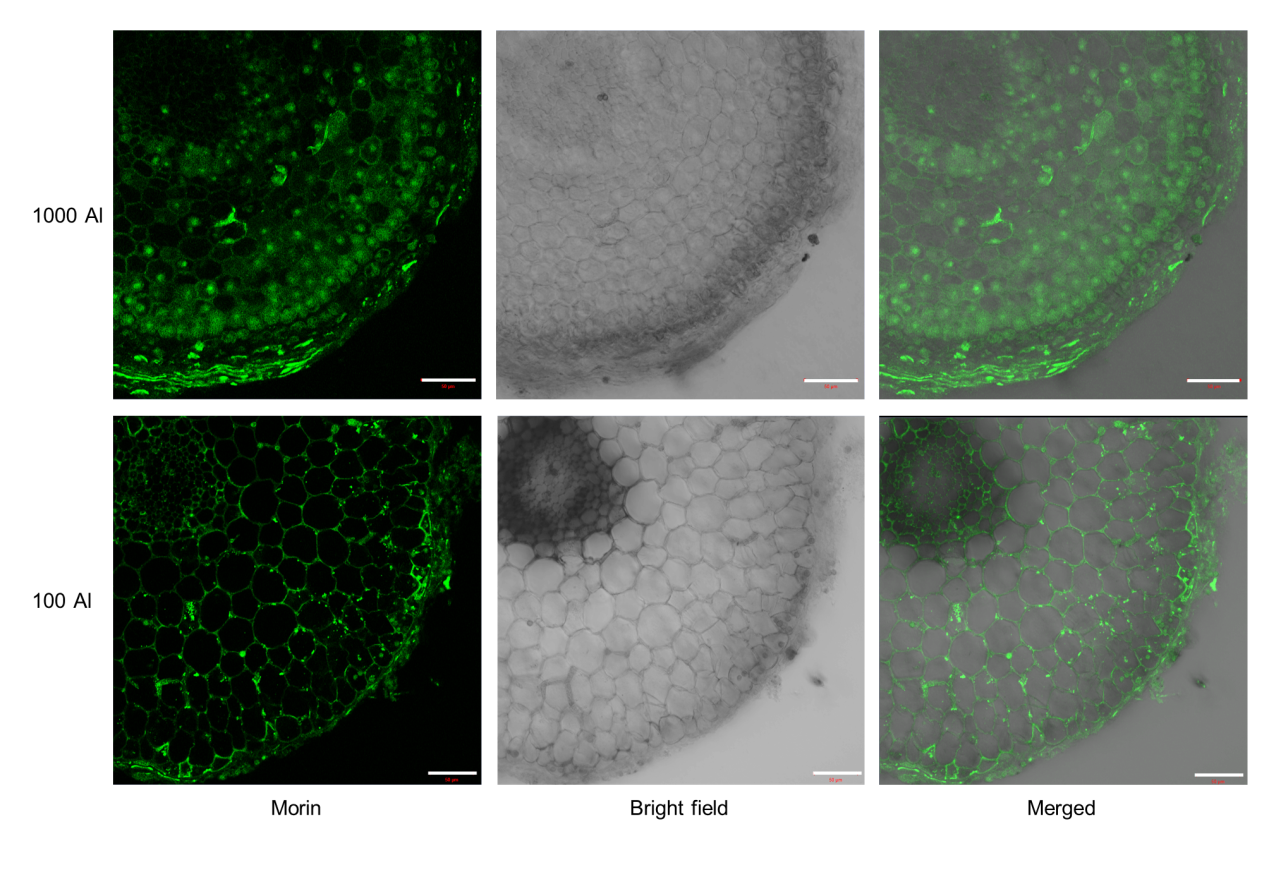


**Supplementary Figure 2. Localization of Al in root apical meristem of tea plant**

Tieguanyin tea plants were grown in solution containing 100 or 1000 µM AlCl_3_ for 30 days. Root transverse sections at 2 mm from the root apex were stained with 100 µM morin solution for 30 min, and photographed by confocal microscopy. Bars = 50 µm.
